# Nurses’ contribution to relatives’ involvement in neurorehabilitation: Facilitators and barriers

**DOI:** 10.1002/nop2.326

**Published:** 2019-07-27

**Authors:** Rikke Guldager, Karen Willis, Kristian Larsen, Ingrid Poulsen

**Affiliations:** ^1^ RUBRIC (Research Unit on Brain Injury Rehabilitation Copenhagen), Traumatic Brain Injury Unit, Department of Neurorehabilitation, Copenhagen University Hospital Rigshospitalet København Denmark; ^2^ Department of Neurosurgery, Copenhagen University Hospital Rigshospitalet København Denmark; ^3^ Department of Learning and Philosophy Aalborg University Aalborg Denmark; ^4^ Allied Health Melbourne Health Melbourne Victoria Australia; ^5^ School of Allied Health La Trobe University Melbourne Victoria Australia; ^6^ Section of Nursing Science, Health Aarhus University Aarhus C Denmark

**Keywords:** healthcare professionals, involvement, nursing, qualitative research, rehabilitation, relatives, traumatic brain injury

## Abstract

**Aim:**

The aim of the present study was to identify possible facilitators and barriers that differently positioned relatives are facing when being actively involved in the rehabilitation process of patients with traumatic brain injury.

**Design:**

A qualitative secondary analysis of data from a qualitative study.

**Methods:**

Data comprised participant observations and semi‐structured interviews with relatives of patients with traumatic brain injury. Data were analysed using a qualitative content analysis.

**Results:**

Three exemplary cases illustrate how relatives’ differential and unequal resources function as facilitators and barriers. Facilitators for involvement are as follows: participating in nursing care situations, the possibility for being present during hospitalization, the relationship with the providers, experience with illness, dedication and proactivity. Contrary, being reactive, non‐participating in nursing care situations, unable to express own wants and needs, and minimal flexibility from workplace are barriers to involvement.

## 1 INTRODUCTION

In contemporary western healthcare systems, individuals are increasingly encouraged to exercise greater control over their own healthcare decisions and there is an expectation that they will be active partners in care (Højgaard & Kjellberg, 2017). However, these expectations are challenged in the case of traumatic brain injury (TBI), a condition associated with high social and economic costs due to long‐term disability and death (Gardner & Zafonte, 2016). Patients with moderate to severe TBI often have physical impairments and disabilities as well as behavioural, social and cognitive sequelae that require long‐term rehabilitation (Benedictus et al., 2010). These impairments affect patients’ participation in the decision‐making process. The role of relatives in involvement therefore becomes vital, as family members become a voice, acting as a proxy to advocate for the patient (Doser & Norup, 2016). How to best involve relatives (who may have varying capacity themselves) in the treatment and care is therefore important and requires support and encouragement from nurses and other healthcare professionals (hereafter called HCPs).

## 2 BACKGROUND

### 2.1 Involvement

Involvement of patients and relatives in western countries is desirable, appropriate and required by health policies; patient involvement is considered important; and this is expressed through political ideas, ethical principles and research in health care (Baines & Regan de Bere, 2018). There are a range of interpretations of how and the extent to which patients and relatives can be involved in their treatment and the decision‐making process.

With their constant presence on the ward, nurses are ideally placed to develop trusting relationships and facilitate the involvement necessary to enable patients and relatives to interact with HCP (Kieft et al., 2014). Thus, involvement is central to nursing practice (Tutton, 2005). Effective involvement requires that nurses have sufficient knowledge of patients’ and relatives’ wish for level of involvement and that patients and relatives are genuinely able to influence decision‐making processes (Vestala & Frisman, 2013). Furthermore, trust and respect between nurses/HCPs and patients/relatives are important for participation in care decision‐making (Vestala & Frisman, 2013).

Facilitating involvement increases the competence of relatives because active involvement of relatives is important for the patient's rehabilitation outcomes (Foster et al., 2012; Oyesanya & Bowers, 2017). Fisher et al. (2017) suggest increasing relatives’ competence to address unmet informational and practical support needs that they might have, but also to benefit individuals with a brain injury by optimizing clinical outcomes.

However, relatives of patients with a TBI often suffer from feelings of anxiety and depression, which may create barriers to involvement. Moretta et al. (2017) found that relatives had a high occurrence of depressive symptoms and anxiety, which emphasizes the need for appropriate psychological and cognitive support therapies for such relatives, also considering their complicated involvement in clinical decision‐making and providing care.

Healthcare professionals, including nurses, may unknowingly be part of the barriers faced by relatives. For example, nurses need to have the competence to support patients’ and relatives’ individual preferences in the best way (Vestala & Frisman, 2013). Keatinge et al. (2002) found that patients considered communication to be the principal barrier to successful partnerships between patients and relatives and concluded that nurses’ lack of communication skills was a barrier to involvement. Lastly, the organizational and work environment can negatively influence patient and relative involvement with lack of time identified as a barrier (Vestala & Frisman, 2013).

### 2.2 Original findings: Three different relative positions

In the research on which this paper is based, the main finding was the identification of three different ideal types appearing as positions occupied by relatives: the warrior, the observer and the hesitant (Guldager, Willis, Larsen, & Poulsen, 2019). The positions illustrate how different positions and related dispositions of relatives influence their strategies in how relatives acted, participated and related to both the patient and the HCPs during rehabilitation.

We were keen to examine how our original findings could translate into nursing practice to optimize the involvement of relatives of patients with TBI. To do this, we wished to focus specifically on illuminating barriers and facilitators for involvement of relatives in the rehabilitation process. Thus, the aim of the present study was to identify the kind of barriers and facilitators to involvement different positioned relatives experience during the rehabilitation process.

### 2.3 Design

In this paper, we report a secondary analysis of data from a broader research project examining how inequality is maintained in a neurorehabilitation setting.

## 3 METHODS

### 3.1 Setting and participants

The study was conducted in a highly specialized rehabilitation department for patients with severe TBI from November 2016–September 2017. Participants were 11 caregivers of nine patients with severe TBI at admission to sub‐acute rehabilitation. Participating relatives were recruited consecutively in collaboration with the interdisciplinary management group of the rehabilitation department. Three exemplary cases of the study participants were chosen, representing one of each of the three relative positions identified in the original study.

### 3.2 Data collection

The method of data collection was participant observation and semi‐structured interviews.

### 3.3 Observations

Guldager et al. attended the formal interdisciplinary meetings where the relatives participated (hereafter called meetings), which were held approximately every 3 weeks. A theory‐based observation recording schedule was constructed, focusing on the interactions between relatives and the HCPs with attention to how the participants were involved. Field notes were taken during the meetings. Twenty‐two meetings were observed. The meetings were scheduled for 30 min and lasted between 20–46 min.

### 3.4 Interviews

A semi‐structured interview guide was constructed, enabling exploration of patients’ and relatives’ experience of the interaction with the HCPs, experience with involvement and information as well as decision‐making during in‐hospital rehabilitation. Initial interviews focused on questions related to the patient's upbringing and educational background, experience with illness and the health system, economic background and social networks, while the follow‐up interviews focused on the relatives’ experience with their own involvement and information as well as decision‐making during in‐hospital rehabilitation. As an example, relatives were asked “what do you experience as positive in the interaction with the HCPs and what challenges do you experience?” A total of 23 interviews, lasting 23–140 min, were recorded digitally and transcribed verbatim.

### 3.5 Secondary data analysis

The mode for this secondary analysis was re‐use of pre‐existing self‐collected qualitative data derived from previous research studies (Heaton, 2008) and is considered as a useful method to find answers to research questions that differ from the questions asked in the original research (Hinds et al., 1997).

Thus, a supplementary secondary analysis of three strategically selected cases was conducted, coding the data in‐depth focusing specifically on the facilitators and barriers three differently positioned relatives are facing when being involved in the rehabilitation process of patients with TBI. The three cases are supported by data from equally positioned individuals from the study, which were evident in the empirical data (Guldager et al., 2019).

The original analysis of 11 interviews and observation field notes was undertaken using a deductive, theory‐based qualitative content analysis following Graneheim and Lundman (2004). The theoretical framework was drawing on Bourdieu's theory of practice, focusing on the conceptual triad of habitus, capital and field. Bourdieu's concept of habitus, the system of dispositions, can be explained as the “ensemble of schemata of perception, thinking, feeling, evaluating, speaking and acting that structures all expressive, verbal and practical manifestations and utterances of a person” (Bourdieu & Bennett, 2010).

Capital comprises three fundamental types: economic capital (e.g., generated wealth, property rights), cultural capital (e.g., educational qualifications, style of speech, skills, taste) and social capital (e.g., network of connections, social obligations) (Lamaison, 1986). Bourdieu suggests that the capacity to leverage capital resources is needed to achieve the best outcomes possible. Finally, all interactions in health care occur within a field of action. A field is a social arena, with its own set of positions and practices and struggles for position, where some individuals are dominant and others are dominated (Bourdieu & Wacquant, 1992). Knowing how the “field” works—referred to as “the rules of the game,” becomes fundamental to being able to engage meaningfully within it.

The concepts have guided the entire research process from construction of the observation recording schedule and the interview guide to data generation, analysis and data interpretation. The methods for the secondary analysis were again a qualitative content analysis, since it is suggested that once conducting qualitative secondary analysis, the same method as the original study should be applied (Heaton, 2008), to stay as close as possible to the original contexts (Andersson et al., 2016). The secondary analysis was conducted in several levels: observation and interviews transcripts were first coded for facilitators and barriers in the rehabilitation process by the first author and then investigator triangulation was applied to reach consensus on the findings to minimize individual researcher bias or personal preference in the analytic process and to ensure the confirmability of the study.

### 3.6 Ethics

The study was conducted according to the principles of the Declaration of Helsinki and registered with the Danish Health Research Ethics Committee (ID 17000765); it was approved by the Danish Data Protection Agency (ID 04346); and data were handled according to its requirements. As the patients were not capable of giving consent, the closest relatives to the patient were contacted by the first author to obtain informed consent. The initial obtained informed consent also covered the re‐using of the data set by ensuring that the data were anonymized, and pseudonyms were used when reporting the findings.

## 4 FINDINGS

This section provides exemplars of high, medium and low degree of relative involvement in the rehabilitation process. The first section presents background information about the three participants and an analysis of possible facilitators and barriers that differently positioned relatives face (Table 1).

**Table 1:** Facilitators of and barriers to being involved in the rehabilitation process

	Anne	Holly	Martin
Facilitators of involvement	Knowledge about patient (former history, family history related to sickness/illness)	Former experience with illness Collaborative‐oriented behaviour Balancing being present and being away Aware of own boundaries	Proactive (action‐ and result‐oriented behaviour) Participating in nursing care situations Personalized information provided Explicit about own wants and needs Maximal flexibility from workplace Possesses cultural skills that align with the providers
Barriers to involvement	Reactive (passive role of involvement) Non‐participant in nursing care situations Standard (not personalized) information provided Unable to express own wants and needs Time‐consuming Minimal flexibility from workplace	Being too collaborative	Being too proactive Lack of respect for family expertise

### 4.1 Participants’ history

The first participant, Anne, is the younger sister to the patient, Arnold, a 52‐year‐old man. Arnold suffered a fall after alcoholic intake and subsequently sustained severe TBI. On admission to the specialized rehabilitation unit, Arnold was in the state of unresponsive wakefulness syndrome (Laureys et al., 2010), meaning that he had no cognitive awareness, only showing reflex movements without response to command (Laureys et al., 2010).

The second participant, Holly, is the mother to the patient, Kevin, aged 29 years. Kevin was a passenger in a traffic accident. On admission, Kevin was in confused state (Laureys et al., 2010) which meant he was disorientated with no day‐to‐day memory, restlessness and no insight in own illness. He did not suffer from any physical impairments (Katz et al., 2009).

The last relative participant, Martin, was a cohabitant male to the patient, Marie, aged 39 years. Marie had been involved in a traffic accident where, as the driver of the car, she was in a head‐on collision with a drunk driver. On admission, Marie was in a minimally conscious state (Laureys et al., 2010), which meant that she showed the first signs of minimal, inconsistent, but reproducible behavioural evidence of self or environmental awareness (Katz et al., 2009; Laureys et al., 2010).

Neither Arnold, Kevin nor Marie was cognitively able to participate in the interdisciplinary meetings (hereafter called meetings) or the interviews. Therefore, Anne, Holly and Martin participated, providing a voice for Arnold, Kevin and Marie, respectively.

### 4.2 Anne

Anne was 51 years old. She grew up in a rural area in a traditional family with both of her parents and six siblings. Anne completed primary school education, followed by a craftsman education. Despite her education, her employment was always in unskilled work. Taking these educational and employment factors into account, it can be argued that Anne's position reflects a low to moderate social position, expressed in and through her choices and preferences. In relation to participation in rehabilitation, Anne's overall position can be characterized as “hesitant”: primarily reactive to decision‐making processes and uncertain of roles (Guldager et al., 2019).

### 4.3 Anne's opportunities for being involved

#### 4.3.1 Knowledge about patient

Anne had thorough knowledge about Arnold's former history and their family history related to sickness/illness, as well as a close relationship with her brother: “Arnold and I, we stick together.” This close relationship positioned Anne as having the expertise to be her brother's voice: “I have always been there for Arnold (laughing) to be honest, yes I have.”

### 4.4 Anne's barriers to being involved

#### 4.4.1 Relation to the HCPs

Anne seemed to have a barrier to being involved which was evident in how she interacted with the HCPs. Her relationship with the HCPs was based on one‐way communication and humility to expertise, trusting that HCPs were well‐equipped to manage Arnold's care. Anne believed in authority and did not challenge HCPs’ decisions.

Anne considered the HCPs as experts in the field of rehabilitation and did not ask questions or raise critical issues in the meetings about treatment and decisions. In the interview, Anne stated that she found it difficult to express her wants and needs and that she had never been able to do that in her life*.*

Anne also stated that she did not know the purpose of the meetings and therefore she was not prepared for what was going to happen and what kind of questions might be relevant to ask. Anne also indicated that she had never asked many questions in her life and that she would adopt a trusting relationship to the nurses, for example, so she could feel confident asking questions. Nonetheless, Anne did not communicate cultural skills and attributes in ways that were recognizable or usable to the HCPs in return for involvement. Neither did she ask questions nor seek information, which may have influenced the nurses’ lack of attention to, or elicitation of, Anne's wants and needs. Anne was not present in the clinic every day, so it was difficult for the nurses to create a relationship with her and get to know her wants and needs. From the observation, it became clear that the nurses did not have much knowledge of Anne and it was evident that the HCPs did not seem to spend much time with her, which had a noticeable effect on the quality of interactions, making involvement difficult. Anne, accustomed to a passive role, therefore presented a practice and behaviour that might be interpreted by the HCPs as satisfied with the level of information and involvement, because she demanded nothing of them. The balance of power between the HCPs and Anne was characterized by an asymmetry, with HCPs having the stronger position.

#### 4.4.2 Non‐participant in nursing care

Despite her close relationship with her brother, Anne considered her role to be that of visitor. For example, when the nurses were in a nursing care situation with Arnold, Anne left the room. She did not perceive she had an option to be involved in his care. As she described one incident herself: “First, there came secretion out and we could see it in the corner of the mouth. And then the secretion came out of the tube. Then we called for the nurses, so they came and cleaned him up.” Anne also expressed concern about being involved, for example, explaining how she asked HCPs for help with repositioning her brother, because his neck was in a bad position with respect to his tracheal tube: “Then the nurse just said, ‘you can reposition him yourself’ and said ‘we do not dare to do that’.”

#### 4.4.3 Possibility of presence

Anne had minimal flexibility from her workplace and was highly dependent on having an income. That meant she had to prioritize her work over visiting her brother. Not being able to visit much made her feel guilty: “I wish I could visit more often, but oh….” As Anne said: “I feel guilty when I cannot be around as much as I would like. It was the same with my husband.”

The timing of the meetings illustrates how the system's structures could erect another barrier to involving Anne. Anne's work conflicted with the timing of one of the meetings at the rehabilitation unit. Although she expressed her wish to be part of the meeting where decisions were made, it was not always possible because of there was no flexibility in terms of time. Anne tried to express her dilemma with the timing of the meeting (10 a.m.), because she had to be at work then, but this did not change anything. When interviewing Anne about how that made her feel, she said: “That made me frustrated, because if the meeting could only be moved a couple of hours later, I would have been able to both take care of my work and attend the meeting.”

#### 4.4.4 Difficulties expressing need for information

Another barrier to involvement related to communication and information. Anne did not express a great need for information during the meetings she was able to attend. Thus, one of the things she recommended to future patients and relatives was: “to be more determined to get more information, though I know it's hard [for the doctor] to say anything about [prognosis]. If only they could say something, anything.” During the meeting, standard information was given by HCPs, but there was a lack of personalized information, which left Anne unsure about her brother's prognosis and future.

Because Anne was passive in her interactions and approach to Arnold's care (being reactive and unable to express her wants and needs), relative involvement was more difficult to achieve.

### 4.5 Holly

Holly was 48 years old. Holly has been married to Fred for more than 30 years; they have three grown‐up children and three grandchildren. Holly grew up in the province, where she is still living. Her father was educated as an accountant and her mother stayed at home, because of a long‐term illness. Holly was college educated and have been employed in sales most of her life except from being a stay‐at‐home mother for 5 years due to fact that her oldest daughter got meningitis and was close to dying. Taking these factors into account, it can be argued that Holly occupies a moderately privileged position in society. In relation to participation in rehabilitation, Holly's overall position can be characterized as “observer”: primarily collaborative with and helpful to, HCPs and concerned to do whatever HCPs direct as being in the best interests of their relative (Guldager et al., 2019).

### 4.6 Holly's opportunities for being involved

#### 4.6.1 Former experience with illness

Holly's experience with her mother and daughter's previous illness, her experience in navigating the health system and a solid belief in that by taking things one thing at a time, gives the best conditions to deal with illness and the rehabilitation process, becomes a strategy she follows in the current course of rehabilitation. This strategy gives Holly the calmness and confidence to be involved in the rehabilitation process with respect to her own boundaries.

#### 4.6.2 Relation to the HCPs

Holly's relationship with the HCPs is characterized by trust, solidarity and loyalty and as a collaboration where she only asks clarifying questions to them if she is in doubt about anything: “I only ask questions or for help if I really need it.” Nevertheless, Holly points out that she does not really know who to ask and if she can ask anyone of the HCP questions. Holly expresses her expectation that HCPs take care of what she perceived to be caring‐related activities such as shaving and cutting nails: “He was lying there and became more and more long‐bearded and I asked if they would shave his beard off. We were told no, we should take care of that ourselves. I think it was very humiliating.” Thus, Holly is aware of her own role as a relative and which nursing care situation she wishes to participate in and which nursing care situations she does not wish to participate in. Holly illustrated one example of this in the interview by telling an example where she was asked to follow her son when he was transferred from one hospital to another: “I said, ‘I won't do it’ I need to get as much sleep as possible, so I won't follow him.”

#### 4.6.3 Possibility of presence

Holly resumed her work during her son's admission at the rehabilitation ward because knowledge from previous experience with illness makes Holly know that she cannot be present all the time in the rehabilitation ward. Holly knows that it is a long process and that she must economize with her powers and resources: “life must be lived despite the accident.” Thus, she tries to create normality outside the hospital.

### 4.7 Holly's barriers to being involved

#### 4.7.1 Being to collaborative

Only one barrier for involvement was identified, which might indicate that Holly's position as an observer may have been harder to identify because they act and align very much with the HCP and their expectations. The only potentially important barrier identified was lack of information. This was evident in how Holly experienced that the HCPs were greeting her when she came to the ward but she did not feel informed about for example her son's day as she thought should have occurred but did not: “Generally when I come, of course they [nurses] greet and such, but there is no… (pause), no one informs me or talk to me. And uh…, I don't know who I can ask, everybody or?”

Holly stated in the interview, how she limited herself asking questions: “I do not ask questions because I don't want to cause any inconvenience.” And further that this was because she wanted to be perceived as a collaborative partner and that she was afraid that Kevin's treatment could be affected if she did not collaborate: “I was afraid that if I said something, they would perceive me as a difficult relative and that it would potentially affect the treatment negatively.” This indicates that Holly was too collaborative, and therefore, her preference for type or amount of information was bypassed, and consequently, there was a lack in the information and communication between the HCPs and the relatives, which becomes a barrier to individually tailored involvement.

### 4.8 Martin

Martin was 47 years old, born and raised in the capital of Denmark and was college educated. After college, Martin was in the military for 2 years and then completed a university degree in export engineer education and was employed as a manager at a large electronic company. Taking these factors into account, Martin's social position could be described as middle or higher. Martin was positioned in the relative position called “the warrior,” characterized as being proactive and fully engaged in decisions about care, directing processes to maximize the benefit for their relative (Guldager et al., 2019).

### 4.9 Martin's opportunities for being involved

#### 4.9.1 Possibility of presence

Martin's workplace provided him with maximum flexibility, enabling him to stay around Marie most of the day and night‐time. Martin's disposition manifested itself throughout the rehabilitation process as engaged and actively involved in Marie's care, developing a range of specialist skills and knowledge during the rehabilitation process such as being legitimized to administer medication: “We have started a new routine. Marie is eating her dinner between 6 and 7 p.m. She uses lots of energy on eating. Afterwards, there is tooth brushing, lip balm, face cream and lots of other stuff.” Martin supplied resources that were appreciated by the professionals: “It is always a pleasure collaborating with good relatives who are present all the time and do so many things” (provider at Marie's meeting).

#### 4.9.2 Relationship with the HCPs

Martin activated strategies, forming an alliance with HCPs, especially the nurses and therapists who were around the patient and relatives all the time. These personal relationships resulted in obtaining informal insider information about future plans for Marie's rehabilitation, outside of the formal meetings: “I already heard it through the grapevine [before it was mentioned at the meeting].” It also resulted in extra time and care: “I notice when they are doing something extra and when they just stick to the routines and things just need to be done and when they really want to do something extraordinary.” The quotes indicate that Martin got personalized information and that Martin saw it as Marie receiving more services and privileges than those in standard rehabilitation. This was underscored in the meetings where Martin's resources and involvement were enhanced by the HCPs, which was evident in how the meetings were dialogue‐based, how the HCPs directed their information to Martin, asking for his opinions and thoughts, and finally, how they encouraged Martin.

#### 4.9.3 Dedication and proactivity

While Martin did not master specific biomedical language, classifications and logic, he was able to draw on his cultural resources to acquire the dominant language and attitudes. In that way, Martin showed awareness of, and adherence to, the “rules of the game” and he embodied a “feel for the game.” He possessed cultural skills that aligned with the HCPs’ who, in turn, perceived him as a “good relative” – an active participant in the rehabilitation process. This was often assessed based on interaction (e.g., emotional relation). For example, Martin used an emotional form of appeal or investment work to achieve sympathy and to optimize Marie's position in the rehabilitation field, so that Marie seemed worth investing in: “Marie is worth investing rehabilitation in,” emphasizing that Marie was not to blame for the accident that she was young, healthy, in good shape, hardworking and vegetarian.

### 4.10 Martin's barrier to being involved

#### 4.10.1 Being too proactive

Although Martin was seen as a resource by HCPs, this could also be a barrier to involvement. For example, Martin explained a situation where his knowledge of how much tube feeding formula Marie could tolerate without vomiting was not recognized: “I simply had to look after them [nurses] so they would realize that they would kill her if she didn't get her food. She was vomiting and it was not working. I think it is a core nursing competency to figure out what the patient can tolerate and then deliver that knowledge to the next nurse, who delivers it to the next… There are potentially three new nurses every day. They simply just couldn't handle it.” In the quotation, Martin touched on how lack of continuity influenced the situation: “Every time a new nurse came, I tried ‘hey, you need to know’ and they said ‘yes, yes, we know a little more’. However, every time a new nurse took care of Marie, she vomited.” Martin's knowledge and reprimanding of the nurses led to the nurses not recognizing his expertise and staying in their own routines despite Martin's knowledge of exactly how much Marie could tolerate. Martin monitored the quality of care, observing every step of the HCPs and trying to influence the selection of staff to protect Marie's physical and emotional safety. It could therefore be a barrier for involvement if Martin perceived nurses as requiring competencies and communication skills, since he was well‐informed, could appear “too much,” could sometimes ask difficult questions and thus risked being judged by HCPs as demanding, critical and as taking up the provider's time (e.g., asking for extra meetings with the consultant even if this was outside standardized rehabilitation).

## 5 DISCUSSION

Using three exemplary cases, we have identified possible facilitators and barriers that differently positioned relatives faced in being involved in the rehabilitation process. Facilitators for involvement are as follows: participating in nursing care situations, being present during hospitalization, good relationship with the providers, experience with illness, dedication and proactivity. Barriers include being reactive, not participating in nursing care situations, inability to express own wants and needs and minimal flexibility from relatives’ workplace regarding time to attend care meetings. The results indicate how Anne, Holly's and Martin's practices, behaviours and strategies became facilitators or barriers influencing their ability to be involved and gain advantages in rehabilitation processes.

### 5.1 Practices, behaviour and strategies

Anne's practice, being reactive and unable to express wants and needs, became a barrier, whereas Martin's strategies of being proactive and explicit about his own wants and needs provided advantages in the field of rehabilitation that values holism, empathy and caring. In the field of rehabilitation, some forms of patient and relative strategies, attitudes and practices are considered more important than others. For example, Martin's proactive attitudes were (mostly) considered by nurses and other HCPs to be appropriate and were credited in the form of praise and recognition.

While Martin's strategies involved being physically present at the meetings, using both empathy and courtesy, using the same language/concepts and in the same logic with the HCPs, Holly's and Anne's strategies were more observing and reactive. Martin's strategies were interpreted differently by the nurses. Most of the nurses collaborated with Martin, letting him be involved in the care. Other nurses did not recognize his expertise and stayed within their professional norms and boundaries of how much and what kind of nursing activities relatives could be involved in, at times making Martin feel excluded as a family expert and in the care of Marie. This tension between using Martin's expertise and maintaining professional expertise could result in not recognizing Martin's expertise.

In contrast to Anne and Holly, Martin could demand being involved and he challenged nurses and HCPs and their decision‐making. The relationship between nurses, other HCPs and Martin was characterized as a power imbalance, but in contrast to Anne, Martin considered himself to be the expert on Marie's medical history. Sohlberg and Mateer suggest that professionals must be willing to “release” their role as the only expert on the team and suggest that relatives’ expertise should be acknowledged and used in decision‐making process (Sohlberg & Mateer, 2013). This could potentially release time for nurses and HCPs that they could invest in relatives with less resources. However, as suggested by Graff et al. (2018), HCPs find it easier to involve those with relatively more resources, because these relatives are able to influence care and treatment, being capable of converting their resources (e.g., cultural) into a dialogue with HCPs. In that way, some relatives can optimize rehabilitation options for patients with TBI (Graff et al., 2018).

### 5.2 Core nursing competencies

Involvement is along with behaviour and attitude, composure, making time for patients, listening and having empathy interpreted as essential nursing competencies (Kieft et al., 2014), and nurses have an important role to play in supporting and balancing relatives’ resources (Reinhard, Given, Petlick, & Bemis, 2008; Sahlsten, Larsson, Plos, & Lindencrona, 2005). However, nurses are not routinely trained to involve patients and relatives. This might indicate an ambiguity between what is expected from the nurses and the actual contribution from the nurses. Ashley (2004) found that it is important to determine whether relatives wish to be involved, whether they are able to be involved and the degree of involvement they wish to have, because not all relatives have the wish, ability or need to be involved (Ashley, 2004). Patients’ and relatives’ right to influence their own health care is laid down in international and national laws (Vahdat, Hamzehgardeshi, Hessam, & Hamzehgardeshi, 2014). Thus, involvement is a requirement and not a choice nurses can make.

Patient‐centred care is one approach keeping the wishes of patients and their relatives at the centre of their care, treatment and support (Kornhaber et al., 2016). This requires that nurses have the ability to form a relationship that elicit patients’ and relatives’ true wishes for involvement (Kornhaber et al., 2016). Thus, nurses need to determine the form that relative involvement should take and take the time needed to embed in the rehabilitation process. Nurses are often the first points of contact for patients and relatives, and the relationship could with advantage been well established at the time of hospitalization (Kieft et al., 2014). In the case of Anne, the long‐lasting and strong tie to her brother seems to be the only facilitator for her involvement in the rehabilitation process, which should be recognized by the nurses, even though it would require time to develop a relationship based on trust and genuine interest and ask questions about Anne and Arnold's former relationship. Nevertheless, time seems to be a key component in developing a trusting relationship. Studies with nurses have found that nurses experience not having enough time to communicate and develop their relationships with patients and relatives and that this hinders the implementation of true, functioning involvement (Dinç & Gastmans, 2012; Sahlsten et al., 2005).

### 5.3 Strengths and limitations

This research has only described and interpreted the relatives' point of view. It could have strengthened the analysis if we had interviewed the nurses about their perspectives on facilitators and barriers in involving the differently positioned relatives in the rehabilitation process. Conducting a secondary analysis of three exemplary cases, strategically selected, to represent relatives with maximum variation enabled us to ensure a valid and nuanced analysis with another perspective than the original analysis.

## 6 CONCLUSION

The results revealed that focusing nursing care on establishing a trusting relation with patients and relatives at the very beginning of hospitalization may contribute to improved practices of involvement in the rehabilitation process, where involvement is based on identification of the relative's needs and where support could thereby be delivered in a more tailored way. Patients and relatives are unequal in terms of socioeconomic resources and this study suggests that nurses should compensate to create equal opportunities for involvement of all relatives, which means addressing the barriers, regardless of the individual's resources and position. This points to the need for more research to identify how to, on an organizational level, handle patients and relatives with fewer resources.

## CONFLICT OF INTEREST

The authors have no conflict of interest to declare.
